# Necrotic Immature Permanent Incisors: Apexification With Calcium Hydroxide and MTA Versus Pulp Revascularization: A Case Report With Long‐Term Follow‐Up

**DOI:** 10.1155/crid/1251988

**Published:** 2026-11-09

**Authors:** Safaa El Omari, Khadija Oumensour, Samira El Arabi, Maria Mtalsi

**Affiliations:** ^1^ Department of Pediatric Dentistry Dental Consultation and Treatment Center (CCTD) Faculty of Dental Medicine Hassan II University of Casablanca Casablanca Morocco uh2c.ac.ma

**Keywords:** apexification, case report, immature permanent teeth, pulp necrosis, pulp revascularization

## Abstract

Pulp necrosis in immature permanent teeth interrupts root development and prevents physiological apical closure, making endodontic management particularly challenging. Apexification with calcium hydroxide or mineral trioxide aggregate (MTA) has long been considered the standard treatment for these teeth by promoting the formation of an apical barrier and achieving periapical healing. However, neither approach allows continued root maturation, leaving thin dentinal walls that may compromise the long‐term prognosis. Regenerative endodontic treatment, including pulp revascularization, has emerged as a biologically based alternative capable of promoting continued root development and reinforcing root structure. This report presents two clinical cases of necrotic immature permanent incisors managed at the Department of Pediatric Dentistry, CCTD Casablanca, Morocco, illustrating calcium hydroxide apexification, MTA apexification, and pulp revascularization. Both treatment approaches resulted in satisfactory clinical and radiographic healing. However, pulp revascularization demonstrated the additional benefit of continued root maturation, whereas apexification provided apical closure without further root development. These cases highlight the indications, advantages, limitations, and clinical outcomes of each therapeutic approach and support the current evidence favoring regenerative endodontic treatment whenever clinical conditions are suitable.

## 1. Introduction

Dental trauma and dental caries are the leading causes of pulp necrosis in children and adolescents [1, 2]. In immature permanent incisors, pulp necrosis arrests root development, resulting in an open apex and posing a major challenge for endodontic treatment [3, 4].

The anatomical characteristics of immature permanent incisors, including a wide‐open apex, short roots, thin dentinal walls, and an unfavorable crown‐to‐root ratio, make conventional endodontic treatment particularly challenging [1, 3, 5]. Achieving an adequate apical seal is difficult, whereas mechanical instrumentation is limited by the fragility of the root walls, increasing the risk of root fracture [6–9].

For many years, calcium hydroxide [Ca(OH)_2_] apexification was considered the gold standard for the management of immature permanent teeth with pulp necrosis [3, 10]. Its alkaline pH (approximately 10.5) provides antibacterial activity and promotes the formation of a calcified apical barrier composed of osteoid‐ or cementoid‐like tissue [3, 4]. However, this technique has well‐documented limitations, including a prolonged treatment duration (6–24 months), the need for repeated intracanal dressing replacement, the risk of coronal reinfection, and progressive weakening of the radicular dentin [4, 7–9].

### 1.1. Haut du formulaire

#### 1.1.1. Bas du formulaire

Mineral trioxide aggregate (MTA) has emerged as a reliable alternative for apexification by enabling the placement of an artificial apical barrier in one or two visits, while providing excellent biocompatibility and superior sealing ability [6, 11–13]. However, neither calcium hydroxide nor MTA promotes continued root maturation; consequently, the treated tooth remains structurally compromised and susceptible to fracture [9, 13, 14].

Regenerative endodontic therapy (RET), also referred to as pulp revascularization, was developed to overcome this fundamental limitation. It is based on effective root canal disinfection, induction of periapical bleeding, and the formation of an intracanal blood clot that serves as a biological scaffold for stem cells from the apical papilla, thereby promoting the restoration of functional pulp‐like tissue and continued root maturation [5, 15–18].

The first case presented herein has particular educational value, as two maxillary permanent incisors (Teeth 11 and 21) from the same patient, affected by the same traumatic injury and at the same stage of root development (Nolla Stage 8), underwent different therapeutic outcomes despite being managed using an identical calcium hydroxide apexification protocol, highlighting the inherent biological variability of treatment response. The second case describes RET with sequential radiographic follow‐up over 36 months, exceeding the minimum follow‐up period of 12–24 months recommended by the American Association of Endodontists (AAE) [18–20].

The aim of this case report is to illustrate, through two clinical cases managed in the Department of Pediatric Dentistry at the Casablanca Dental Consultation and Treatment Center (CCTD), three therapeutic approaches for the management of immature permanent teeth with pulp necrosis—calcium hydroxide apexification, MTA apical barrier, and RET—and to discuss their indications, advantages, and limitations in light of the current evidence and recommendations from international scientific societies [2, 5, 18].

## 2. Clinical Case No. 1—Apexification: Ca(OH)_2_ versus MTA Apical Plug

A 10‐year‐old boy with no relevant medical history was referred to the Department of Pediatric Dentistry, CCTD, for the management of recurrent sinus tracts associated with the maxillary central incisors (Teeth 11 and 21). His dental history revealed a previous traumatic injury resulting from a fall. Written informed consent was obtained from the patient′s parents before treatment in accordance with current ethical guidelines.

Clinical examination revealed complicated crown fractures (Ellis Class III) involving both maxillary central incisors, defective temporary restorations, and active sinus tracts adjacent to the corresponding apices. Based on the clinical and radiographic findings, a diagnosis of pulp necrosis was established.

Periapical radiographs revealed open immature apices in both teeth, corresponding to Nolla Stage 8, together with periapical radiolucencies and enlarged root canals (Figure 1). The roots exhibited thin, parallel dentinal walls. Radiographically, the apical diameter was greater in Tooth 11 than in Tooth 21.

**Figure 1:**
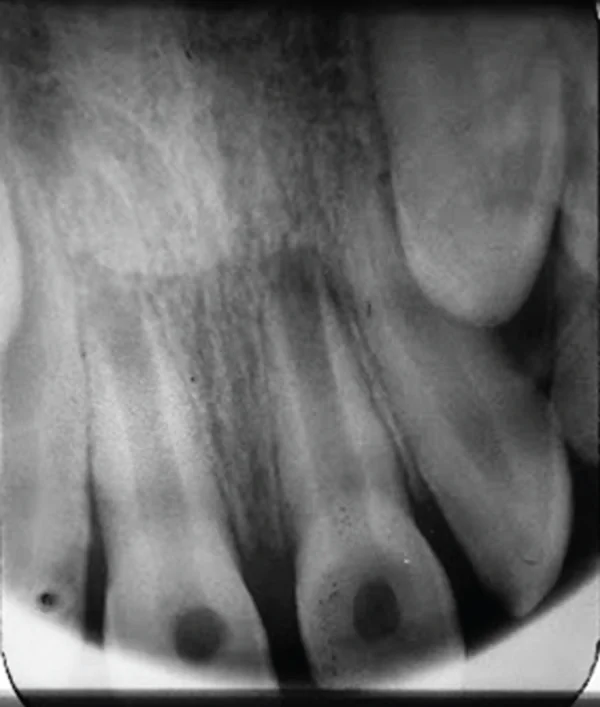
Baseline periapical radiograph (Day 0) showing immature open apices of teeth 11 and 21 (Nolla Stage 8) associated with bilateral periapical radiolucencies.

Under rubber dam isolation, the access cavities of teeth 11 and 21 were refined, followed by root canal debridement without aggressive mechanical instrumentation. The canals were irrigated with 2% sodium hypochlorite (NaOCl) using a closed‐end irrigation needle positioned 2 mm short of the apical foramen, followed by saline irrigation. Copious intracanal exudate was observed in both canals during this visit (Figure 2), indicating persistent periapical exudation. Calcium hydroxide [Ca(OH)_2_] was placed as an intracanal medicament, and the access cavities were sealed with glass ionomer cement.

**Figure 2:**
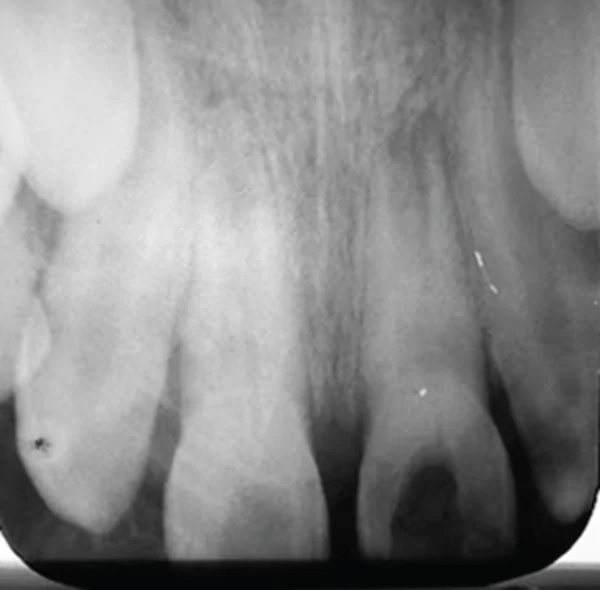
Baseline periapical radiograph (Day 0) showing Teeth 11 and 21 with periapical radiolucencies; copious intracanal exudate was observed clinically during root canal drying.

At the subsequent appointment, in the absence of residual intracanal exudation, the root canals of teeth 11 and 21 were obturated with calcium hydroxide [Ca(OH)_2_]. The quality of the canal filling was confirmed radiographically (Figure 3).

**Figure 3:**
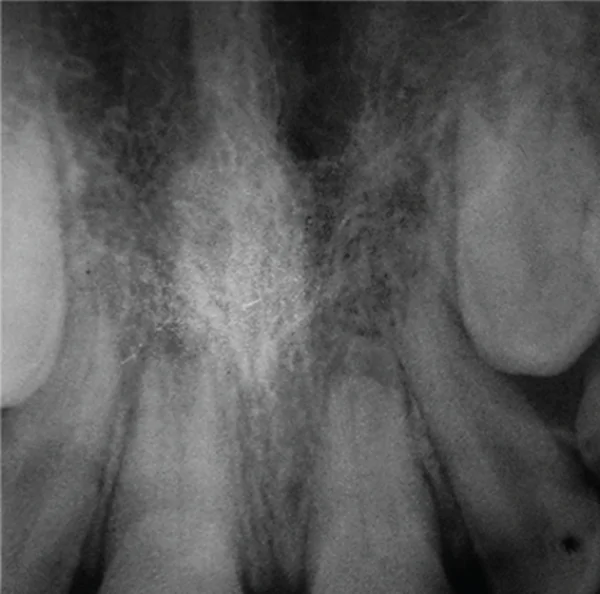
Periapical radiograph obtained at the 15‐day follow‐up showing dense intracanal calcium hydroxide [Ca(OH)_2_] dressing in Teeth 11 and 21.

At the 6‐month follow‐up, periapical radiographs showed a reduction in the periapical radiolucencies associated with both teeth. Initial formation of a thin calcified apical barrier was observed in Tooth 11, whereas no apical barrier was visible in Tooth 21 (Figure 4). Calcium hydroxide [Ca(OH)_2_] was renewed in both root canals.

**Figure 4:**
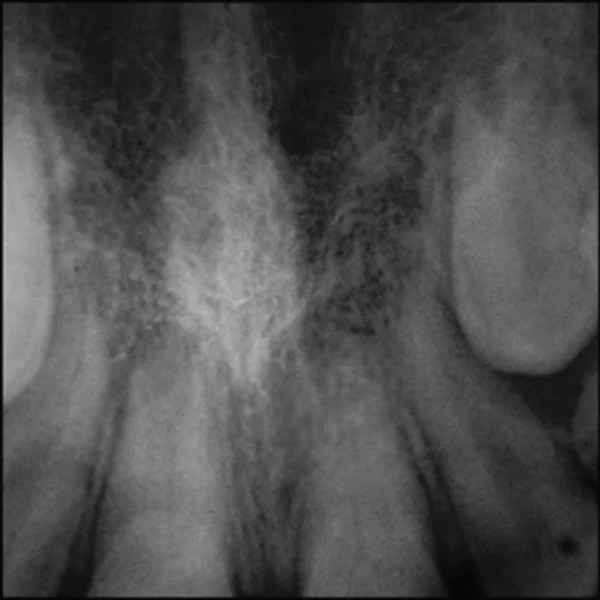
Six‐month follow‐up periapical radiograph showing regression of the periapical radiolucencies and initial formation of a calcified apical barrier in Tooth 11 only.

At the 9‐month follow‐up, periapical radiographic assessment using a gutta‐percha master cone confirmed the presence of a newly formed calcified apical barrier in Tooth 11 (Figure 5). In contrast, Tooth 21 continued to exhibit an open apex despite 9 months of calcium hydroxide [Ca(OH)_2_] therapy, although it had presented with comparable initial conditions and a narrower apical diameter than Tooth 11. Consequently, a 4‐mm MTA apical barrier (ProRoot MTA; Dentsply Sirona, York, Pennsylvania, United States) was placed in tooth 21 during the same appointment (Figure 6).

**Figure 5:**
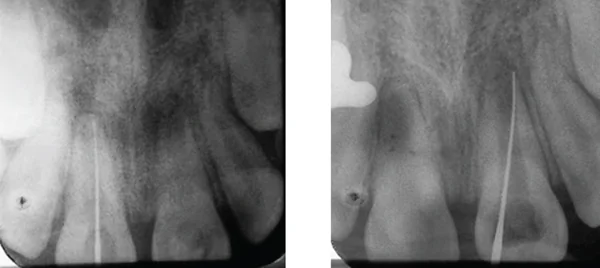
Nine‐month follow‐up periapical radiograph with a gutta‐percha master cone confirming the formation of a calcified apical barrier in Tooth 11, whereas Tooth 21 continued to exhibit an open apex.

**Figure 6:**
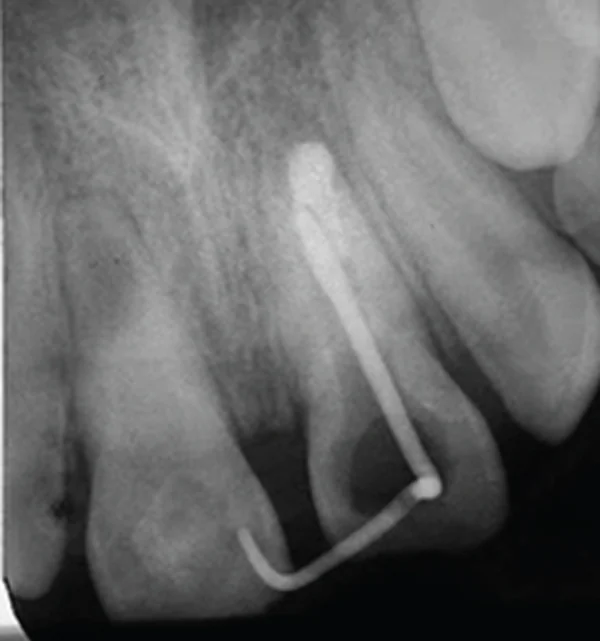
Nine‐month follow‐up periapical radiograph showing placement of a 4‐mm mineral trioxide aggregate (MTA) apical barrier in tooth 21.

Root canal obturation was performed in teeth 11 and 21 using gutta‐percha and the lateral condensation technique, followed by coronal restoration with direct composite resin (Figure 7). No pathological clinical signs were observed at the immediate postoperative follow‐up.

**Figure 7:**
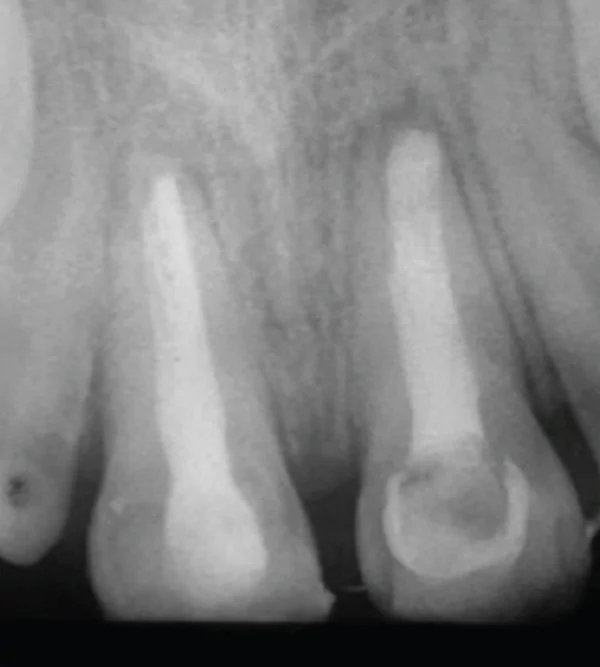
Periapical radiograph showing definitive gutta‐percha root canal obturation of Teeth 11 and 21 after apexification, with satisfactory coronal sealing.

The chronological summary of the clinical and radiographic follow‐up for Case 1 is presented in Table 1.

**Table 1:** Chronological summary of follow‐up, Case No. 1.

Time point	Tooth 11	Tooth 21	Figure
Baseline	Open apex (Nolla Stage 8)	Open apex (Nolla Stage 8)	Figure 1
Baseline to 15‐day follow‐up	Intracanal Ca(OH)_2_ dressing (initial exudate present)	Ca(OH)_2_ intracanal dressing (copious initial exudate)	Figures 2 and 3
6‐month follow‐up	Periapical healing. Initial calcified apical barrier	Reduced periapical radiolucency. Persistent open apex	Figure 4
9‐month follow‐up	Calcified apical barrier confirmed (gutta‐percha master cone)	Persistent open apex →4‐mm MTA apical barrier	Figures 5 and 6
Definitive treatment	Definitive root canal obturation and coronal restoration	Gutta‐percha obturation + coronal restoration	Figure 7

## 3. Clinical Case No. 2—Pulpal Revascularization of Tooth 21

An 8‐year‐old girl with no relevant medical history was referred to the CCTD for the management of a vestibular abscess associated with Tooth 21 following iatrogenic dental treatment. Written informed consent was obtained from the patient′s parents before treatment.

Clinical examination revealed an active vestibular abscess associated with Tooth 21 and a defective temporary restoration. No pathological tooth mobility or tenderness to percussion was observed.

Periapical radiographic examination revealed an open immature apex with an apical diameter of 1.1 mm, thin and parallel dentinal walls consistent with Nolla Stage 8, and a marked periapical radiolucency (Figure 8). The root canal appeared enlarged throughout the entire root length, indicating arrested root development.

**Figure 8:**
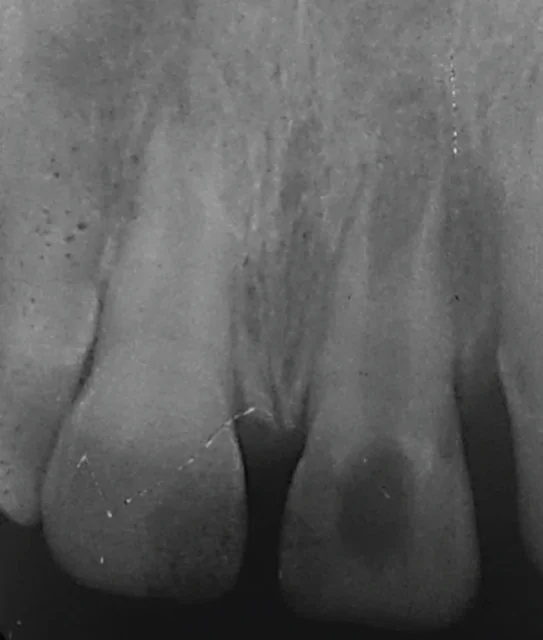
Baseline periapical radiograph (Day 0) showing Tooth 21 with an immature open apex (apical diameter: 1.1 mm), consistent with Nolla Stage 8, and an extensive periapical radiolucency.

Based on the clinical and radiographic findings, a diagnosis of pulp necrosis with chronic apical periodontitis in an immature permanent tooth was established.

Considering the stage of root development, the thin dentinal walls, arrested root development, and an apical diameter of 1.1 mm, regenerative endodontic treatment was selected as the first‐line therapeutic approach. An apical diameter of ≥ 1.1 mm facilitates the ingrowth of stem cells from the apical papilla and increases the likelihood of successful regenerative treatment by 18%–34%.

Because of the presence of an active abscess, systemic amoxicillin (50 mg/kg/day in two divided doses for 7 days) was prescribed as first‐line therapy.

Under rubber dam isolation, the access cavity of Tooth 21 was refined, followed by root canal debridement without mechanical instrumentation of the canal walls, in accordance with the AAE recommendations. The canal was irrigated with 2% sodium hypochlorite (NaOCl) using a closed‐end irrigation needle positioned 2 mm short of the apical foramen, followed by sterile saline. Calcium hydroxide [Ca(OH)_2_] was placed as a temporary intracanal medicament for 1 week (Figure 9).

**Figure 9:**
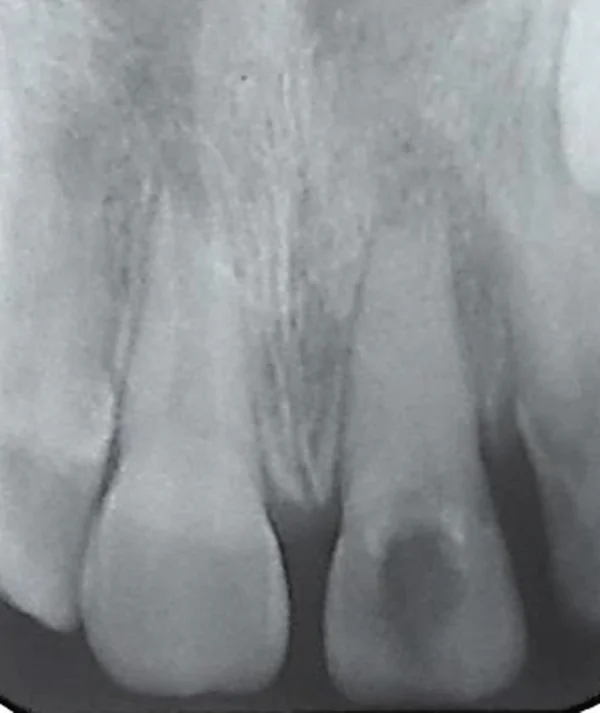
Baseline periapical radiograph showing placement of an intracanal calcium hydroxide [Ca(OH)_2_] medicament in Tooth 21.

Under rubber dam isolation, the intracanal calcium hydroxide was removed by gentle irrigation. Local anesthesia without a vasoconstrictor (3% mepivacaine) was administered to preserve the local vasodilation required for the formation of a stable intracanal blood clot. Intracanal bleeding was then induced using manual K‐files extended 2 mm beyond the working length with slow rotational movements (Figure 10). Once a stable blood clot had formed up to the cervical third and hemostasis had been confirmed, a 15‐minute waiting period was observed before proceeding.

**Figure 10:**
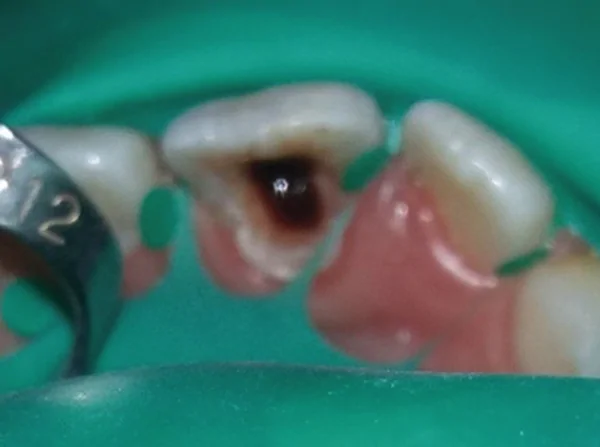
Controlled induction of intracanal bleeding resulting in the formation of a stable blood clot.

A 3–4 mm MTA plug (ProRoot MTA, Dentsply Sirona) was placed in the cervical third of the root canal in direct contact with the blood clot (Figure 11). The access cavity was hermetically sealed with glass ionomer cement.

**Figure 11:**
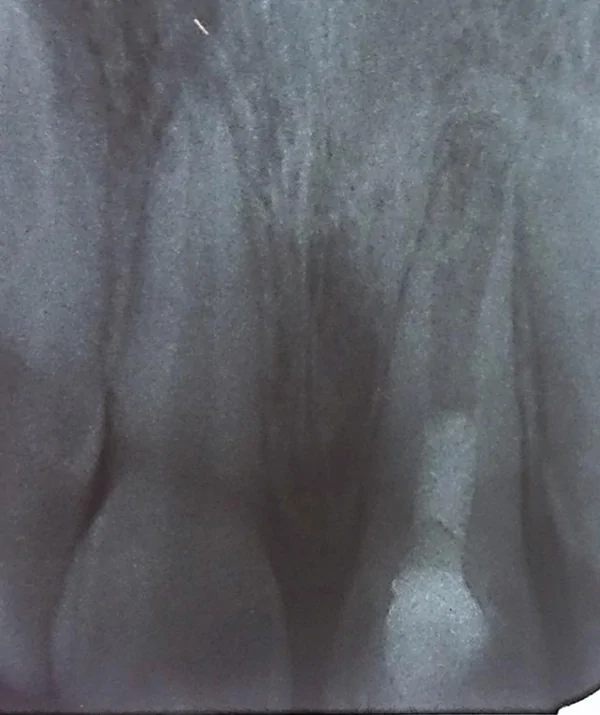
Periapical radiograph showing placement of a 3–4 mm cervical mineral trioxide aggregate (MTA) plug over the blood clot in Tooth 21.

Sequential periapical radiographs documenting the evolution of Tooth 21 at 6, 12, 24, and 36 months following regenerative endodontic treatment are shown in Figure 12.

**Figure 12:**
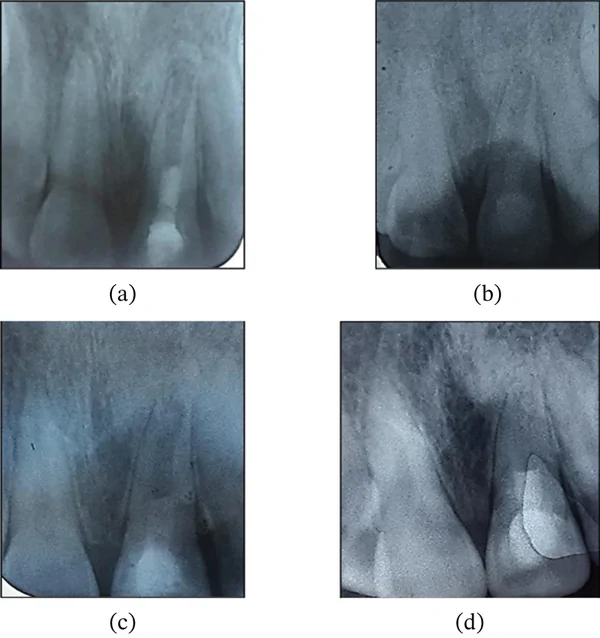
(a–d) Sequential periapical radiographs of Tooth 21 following regenerative endodontic treatment: (a) 6 months; (b) 12 months; (c) 24 months; and (d) 36 months.

The chronological summary of the radiographic follow‐up for Case 2 is presented in Table 2.

**Table 2:** Radiographic follow‐up summary for Case 2.

Time point	Radiographic findings	Interpretation	Figure
6 months	Continuous periodontal ligament space with an intact lamina dura	Periapical healing	Figure 12a
12 months	Apical barrier formation	Initiation of apical closure	Figure 12b
24 months	Complete apical closure	Apical closure associated with increased root length	Figure 12c
36 months	Partial canal obliteration with thickening of the root canal walls	Continued root development indicating regenerative endodontic success	Figure 12d

## 4. Discussion

Endodontic management of necrotic immature permanent teeth remains a clinical challenge due to the presence of a wide‐open apical foramen, thin and fragile dentinal walls, and an unfavorable crown‐to‐root ratio. Currently, three therapeutic approaches are available, whose indications and levels of evidence have been defined by the AAE, the European Society of Endodontology (ESE), and the European Academy of Paediatric Dentistry (EAPD) [2, 5, 18].

Apexification with calcium hydroxide [Ca(OH)_2_] represents the earliest documented treatment approach [3, 10]. Its mechanism is based on hydroxyl ion release, resulting in an alkaline environment (pH ≈10.5) that promotes bacterial protein denaturation, whereas calcium ions stimulate mineralization through alkaline phosphatase activity, leading to the formation of an osteoid‐ or cementum‐like apical barrier [3, 4, 12]. In our first case, Tooth 11 achieved apical barrier formation within 9 months, consistent with the treatment duration reported in the literature (5–20 months; mean: 12 ± 7 months) and success rates ranging from 74% to 100% [4, 14, 21].

Recent updates in institutional recommendations provide the appropriate context for interpreting our cases. The AAE progressively revised its clinical recommendations (2013, 2016, 2018, and 2021), ultimately no longer listing calcium hydroxide apexification as a standalone treatment option for necrotic immature permanent teeth [18]. The EAPD has adopted a similar position, whereas the ESE, in its position statement on revitalization procedures, formalized a standardized regenerative protocol recognizing revitalization as a valid alternative to conventional apexification [5]. Kahler et al. [9] emphasized that immature permanent teeth undergoing prolonged calcium hydroxide apexification are at an increased risk of cervical root fracture. Although this risk has traditionally been attributed to alkaline‐induced alterations of the dentinal collagen matrix, current evidence suggests that the inherent structural weakness and thin dentinal walls of immature teeth may be the principal contributing factors [7–9].

The most informative finding from this first case was the divergent response of Teeth 11 and 21 to an identical treatment protocol. Despite sharing the same etiology, Nolla stage, and frequency of calcium hydroxide [Ca(OH)_2_] replacement, Tooth 21 failed to demonstrate apical barrier formation after 9 months. This observation is particularly noteworthy because Tooth 21 initially exhibited a narrower radiographic apical diameter than Tooth 11, although a smaller initial apical width has been suggested to favor faster apical barrier formation [3]. However, our findings are consistent with those of Kleier and Barr et al. [21], who demonstrated that the extent of the periapical lesion may influence and delay apexification. These results further support the observations previously reported by our group Boufdil et al. [22], highlighting the unpredictable response of necrotic immature permanent incisors to calcium hydroxide apexification and emphasizing the importance of regular radiographic monitoring to guide treatment modifications throughout the healing process.

Given the absence of apical barrier formation in Tooth 21 after 9 months, placement of an MTA apical plug was clinically justified, in accordance with the current recommendations of the AAE [18] and the ESE [5]. Limiting prolonged exposure to calcium hydroxide [Ca(OH)_2_] is also considered a preventive strategy to preserve dentinal integrity, as extended intracanal medication has been associated with deterioration of the mechanical properties of radicular dentin and an increased risk of root fracture [7–9]. The systematic review and meta‐analysis by Panda et al. [14], which included 32 studies, demonstrated favorable outcomes of MTA apexification compared with calcium hydroxide, with some of the included studies reporting success rates approaching 100% after 12 months of follow‐up. More recent bioceramic materials, such as Biodentine, represent promising alternatives to MTA, offering shorter setting times and a reduced risk of discoloration while achieving comparable clinical outcomes according to recent randomized controlled trials [23, 24]. However, the main limitation of all apexification procedures remains unchanged: They create an artificial apical barrier without promoting true continued root development, leaving the immature tooth structurally compromised over the long term [3, 13, 14].

The regenerative endodontic protocol applied in the second case followed the six key steps outlined in the international expert consensus on regenerative endodontic procedures [25], together with the joint recommendations of the AAE [18] and the ESE [5]. These steps include minimal instrumentation of the canal walls, irrigation‐based disinfection, intracanal medication, induction of intracanal bleeding with blood clot formation, placement of a bioceramic barrier, and hermetic coronal restoration. The use of 2% sodium hypochlorite was consistent with the concentration range (1.5%–3%) recommended by both the AAE and the ESE for regenerative endodontic procedures [5, 18].

The selection of intracanal medicaments in regenerative endodontic procedures remains an area of ongoing debate. Three main options are currently considered: calcium hydroxide [Ca(OH)_2_], triple antibiotic paste (TAP; ciprofloxacin, metronidazole, and minocycline), and double antibiotic paste (DAP; ciprofloxacin and metronidazole) [18, 26]. In recent years, increasing interest has been directed toward DAP because it avoids the use of minocycline while maintaining antimicrobial effectiveness [27]. The inclusion of minocycline in TAP is associated with clinically significant coronal discoloration [9, 28], whereas high concentrations of antibiotic pastes may adversely affect the viability of stem cells of the apical papilla in a dose‐dependent manner [16, 17]. Consequently, the AAE [29] recommended the use of DAP or replacement of minocycline in anterior teeth undergoing regenerative procedures. In the present case, Ca(OH)_2_ was selected as the initial intracanal medicament because of its favorable biocompatibility with stem cells of the apical papilla, well‐documented antimicrobial properties, and absence of discoloration risk in the anterior region of an 8‐year‐old patient. This approach is consistent with current recommendations, which consider Ca(OH)_2_ and DAP as preferred alternatives to TAP for regenerative procedures in anterior teeth [5, 18, 27].

The intracanal blood clot acts as a biological scaffold enriched with growth factors, including platelet‐derived growth factor (PDGF), transforming growth factor‐*β* (TGF‐*β*), and vascular endothelial growth factor (VEGF), which promote the migration and differentiation of stem cells from the apical papilla, thereby supporting the regeneration of pulp‐like tissue and continued root development [15, 26, 30]. In our case, the 36‐month sequential radiographic follow‐up documented the four sequential radiographic changes observed during successful regenerative healing. These findings are consistent with recent evidence. The meta‐analysis published in the *British Dental Journal* [31], which included nine randomized controlled trials, reported an overall success rate of 90% for regenerative endodontic procedures (95% CI: 83%–94%), with recovery of pulp sensibility in 56% of cases and continued root development. Furthermore, the 5‐year prospective study by Jothish et al. [19], involving 100 patients, reported a tooth survival rate of 92% following regenerative treatment. More recently, the systematic review by Anas et al. [32] further supported the superiority of regenerative endodontic procedures over apexification, particularly with respect to root lengthening and increased dentinal wall thickness.

The partial root canal obliteration observed at the 36‐month follow‐up is consistent with intracanal calcification associated with regenerative endodontic procedures, as described by Song et al. [33], who reported a prevalence of 62.1% among 29 treated cases. More recent evidence further supports these findings and suggests that the use of calcium hydroxide [Ca(OH)_2_] as an intracanal medicament may represent a contributing factor, with calcification rates of 76.9% compared with 46.2% when antibiotic‐based medicaments were used [20]. Although this outcome does not appear to interfere with the resolution of apical periodontitis, it may complicate potential future endodontic retreatment and should therefore be anticipated and discussed with parents during the treatment planning process [20, 33, 34].

The outcomes observed in these two cases, interpreted in light of the current recommendations of the AAE [18], the ESE [5], and the EAPD [35], confirm that all three therapeutic approaches may achieve favorable periapical healing. However, regenerative endodontic treatment remains the only approach capable of promoting continued root development and increasing root structural maturity, thereby improving the long‐term mechanical prognosis of immature teeth, an advantage that conventional apexification procedures cannot provide [31, 32, 36, 37]. Therefore, regenerative endodontic procedures should be considered the preferred first‐line treatment for necrotic immature permanent teeth with arrested root development when adequate apical dimensions, effective infection control, and patient cooperation are achieved [5, 18, 25]. When regeneration is not feasible, MTA‐ or Biodentine‐based apexification represents a reliable alternative [13, 14, 23]. Ultimately, treatment selection should remain individualized, considering the degree of root maturation, apical diameter, esthetic considerations, and operator expertise [5, 18].

## 5. Conclusion

These two cases demonstrate that favorable periapical healing can be achieved with different treatment approaches. However, regenerative endodontic treatment remains the only modality capable of promoting continued root development, offering the potential for improved long‐term structural integrity compared with conventional apexification techniques. Therefore, it should be considered the preferred first‐line treatment for necrotic immature permanent incisors when clinical conditions are favorable.

The first case highlights the unpredictable biological response to calcium hydroxide [Ca(OH)_2_] apexification, even among teeth with similar initial characteristics, and emphasizes the importance of timely reassessment and transition to an MTA‐based approach when apical barrier formation fails to occur. The second case illustrates, over a 36‐month follow‐up period, the potential of regenerative endodontic treatment to promote continued root maturation in young patients, with progressive canal obliteration representing a radiographic outcome that does not compromise clinical success but warrants long‐term follow‐up.

Although limited by the small number of cases, this report does not allow statistical conclusions. Nevertheless, it emphasizes the importance of individualized treatment planning and the clinician′s ability to master the three available therapeutic approaches. Further prospective studies with larger cohorts are needed to optimize regenerative protocols, refine case selection criteria, and better define the respective roles of calcium hydroxide and DAP as intracanal medicaments in regenerative endodontic procedures.

## Funding

No funding was received for this manuscript.

## Ethics Statement

This case report was conducted in accordance with the ethical principles of the Declaration of Helsinki and institutional requirements.

## Consent

Written informed consent was obtained from the patient′s mother and legal guardian for the publication of the patient′s clinical details and photographs in this case report.

## Conflicts of Interest

The authors declare no conflicts of interest.

## Data Availability

The data that support the findings of this study are available on request from the corresponding author. The data are not publicly available due to privacy or ethical restrictions.
